# Observation of intracellular bacterial communities in urinary sediment using brightfield microscopy; a case report

**DOI:** 10.1186/s12894-020-00661-y

**Published:** 2020-07-06

**Authors:** Carlos Martínez-Figueroa, Karen Cortés-Sarabia, Luz del Carmen Alarcón-Romero, Hilda Guadalupe Catalán-Nájera, Micaela Martínez-Alarcón, Amalia Vences-Velázquez

**Affiliations:** 1 Laboratorio de Inmunobiología y Diagnóstico Molecular, Facultad de Ciencias Químico Biológicas, Universidad Autónoma de Guerrero, Av. Lázaro Cárdenas s/n, University City, C.P.39090 Chilpancingo, Guerrero Mexico; 2 Servicio de urgencias, Laboratorio clínico, Clínica Hospital ISSSTE, Iguala, Guerrero Mexico

**Keywords:** Urinary tract infections, *Escherichia coli*, Intracellular bacterial communities, Urinary sediment, Case report

## Abstract

**Background:**

Urinary tract infections (UTIs) are usually related to the presence of *Escherichia coli*, a microorganism that adopts an intracellular life-style during the pathogenesis of cystitis. Evidence of the underlying mechanism in urothelial cells from urine samples has been reported. However, intracellular communities have not yet been described in squamous cells in fresh samples stained with Sternheimer-Malbin method, thus, we have provided these descriptions in this case report.

**Case presentation:**

Number 1 was a male patient with symptoms of UTI, his urinalysis revealed hematuria and nitrites. In the urine sediment, we found urothelial cells with internal endosomes filled with short rods. Case number 2, female patient with recurrent UTI by *E. coli,* her urinalysis showed positive nitrites, glycosuria, bacteriuria and squamous cells with endosomes filled with short rods. Both patients were positive for *E. coli* isolation.

**Conclusions:**

These case reports provide evidence of the presence of intracellular bacterial communities in urothelial and squamous cells (not previously reported) in fresh urine samples stained with Sternheimer-Malbin using brightfield microscopy. The clinical impact and pathogenic mechanisms involved in the invasion of the squamous epithelium need further investigation.

## Background

*Escherichia coli* is the main etiological agent associated with urinary tract infections (UTIs). This microorganism is perfectly adapted to the environment of the urinary system. During the pathogenesis of cystitis, this bacteria use the intracellular route to form an intracellular biofilm, named as intracellular bacterial community (IBC), that has been studied in both in vivo and in vitro models [1, 2]. IBCs are formed in superficial urothelial cells, they go through several maturation stages leading to biofilm reorganization before differentiating into the filamentous and coccoid morphotypes commonly found in the late intracellular community [2, 3]. Only a few cases on the presence of IBCs in urothelial cells of patients with UTI have been reported. Here, we report two new cases involving not only urothelial cells but also squamous cells.

## Case presentation

### Patients information

#### Case number 1

A 50-year-old male patient, without any background of chronic or infectious diseases, urinary stones, or anatomical abnormalities, came to the hospital’s emergency room with pelvic pain but no fever, vomiting or chills. Reported symptoms included dysuria, polaquiuria, hematuria and foul smelling urine.

#### Case number 2

A 64-year-old patient, with a background of diabetes mellitus, hypertension and hyperthyroidism, came to the bacteriological service area of the hospital’s emergency room for controlling a recurrent UTI. No additional symptoms were reported. During the follow up, the patient showed a few episodes of healing and relapse.

### Diagnostic assessment

For urinalysis, 10 mL of urine were aliquoted in a 16 × 150 mm tube. Chemical tests were performed using test strips (SPINREACT URIN-10). After chemical testing, urine was centrifuged for 5 min at 450 g, supernatant was aspirated using a pipette until 0.5 mL was left and 50 μL of Sternheimer-Malbin dye was added. The Sternheimer-Malbin stain is a supravital dye composed by safranin O and cristal violet, is used to contrast the formed elements and to stain structures that have lost their vitality. Finally, sediments were examined under a brightfield microscope at 40× magnification.

#### Case number 1

**Fig. 1:**
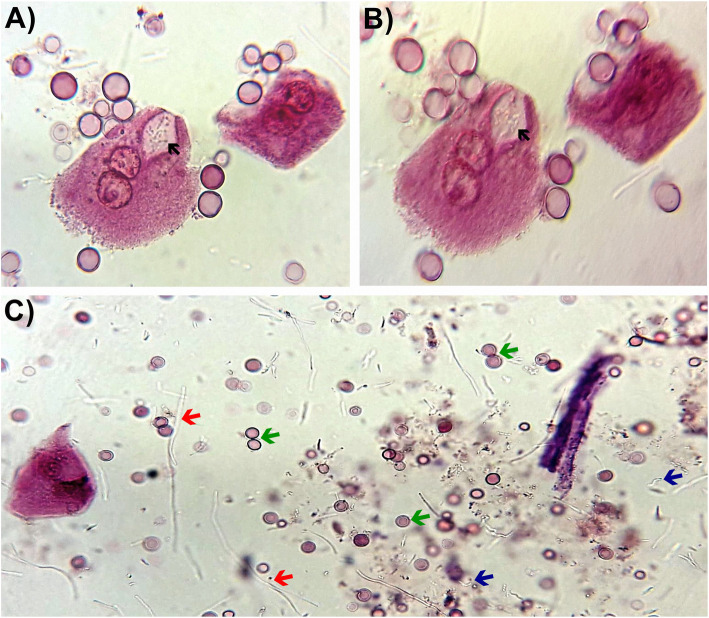
Representative image of the urinary sediment of a patient with *E. coli* infection. **a** Clear field microscopy and **b** clear field microscopy with cylinder-type illumination images. Note the presence of urothelial cells with bacteria inside an endosome (black arrows). **c** Bright field microscope image showing different bacterial morphotypes, short (blue arrows) and long (red arrows) rods, and hematuria (green arrows). Images are at a 40× magnification

Nitrites and hematuria (> 100 erythrocytes/field) were confirmed by chemical and microscopic analyses of the patient’s urine sample. In the sediments, superficial urothelial cells, along with scarce squamous cells, monohydrate calcium oxalate, and moderate bacteriuria including two bacterial morphotypes (short rods and filamentous forms) were observed, whereas no-leukocytes were found. In the interior of an endosome from a superficial urothelial cell, we observed the presence of moving short rod bacteria (Fig. 1) (supplementary material 1).

**Additional file 1.**

#### Case number 2

**Fig. 2:**
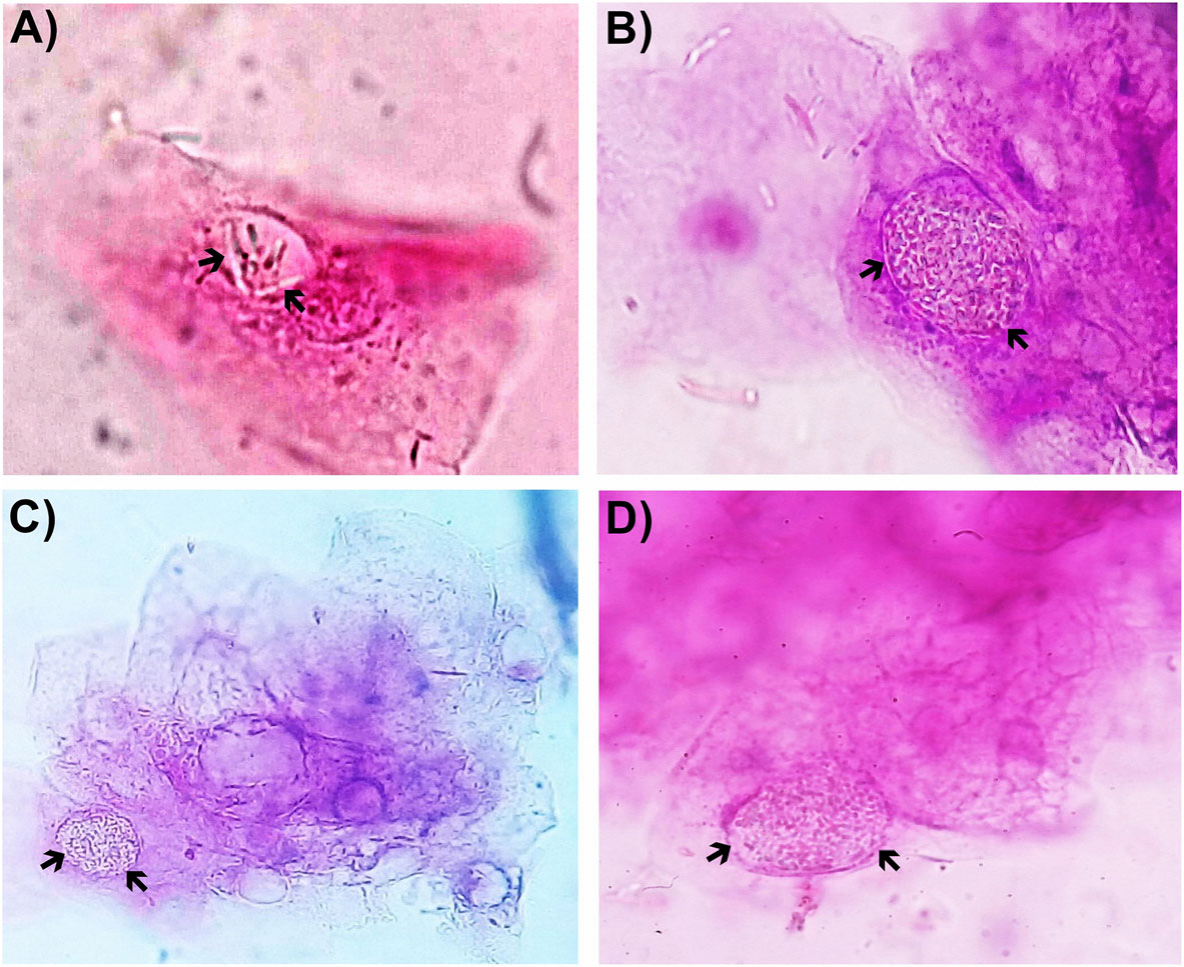
Intracellular bacteria in the urinary sediment. **a**-**d** Bright field microscope image showing squamous cells with bacteria inside an endosome (black arrows). The sample was stained using the Sternheimer-Malbin method. The image is at 40× magnification

Nitrites and glycosuria were confirmed by chemical analysis of the patient’s urine sample. In the sediment test, we observed bacteriuria, leukocyturia (5–10/field), and squamous cells with endosome-containing bacteria. Moving along different focus planes in the microscope, we were able to determine that short rods were in the interior of the cells and not in their surface. This location was confirmed in at least ten squamous cells along the sediment preparation (Fig. 2).

### Diagnostic

For both cases, we isolated *E. coli* with colony counts greater that > 100,000 UFC/mL.

## Discussion and conclusions

IBC formation is a protective mechanism of invasion of the bladder by *E. coli*. It has been reported that biofilm formation is related with the evasion of the host innate immune response, antibiotic resistance, and urinary tract persistence [3, 4]. However, there are just a few reports about the presence of IBCs in the urine of patients with cystitis. Rosen et al. (2007), reported the existence of IBC-like structures in women with cystitis, these structures were similar to those exfoliated intracellular communities observed in the urine of mice with cystitis, and that this pathogenic route could be implicated in the recurrence of the infection [5]. Whereas, Robino et al. (2014) found IBCs in 36.8% of a population of children with urinary tract infections by *E. coli*. The presence of these IBCs was associated with recurrent infections in patients without morphological or structural abnormalities in their urinary tracts [6].

The squamous epithelium is found in the bladder trigone and the urethra in both sexes. The presence of IBCs in squamous cells could indicate that there is an infection in any of these sites, an infection that may have not been previously described. In the contrary, IBC formation in urothelial cells is generally related to know infections. Consequently, further investigation to establish pathogenic mechanism mediating IBC formation in squamous cells and its contribution to the development of cystitis is needed [2, 7].

According to our findings, the contributions of this study to the field of UTIs are listed as follows: A) The presence of IBCs in fresh samples can be determined using Sternheimer-Malbin stain and brightfield microscopy, instead of electronic microscopy of confocal microscopy and Giemsa stain, as reported previously; B) IBC may form in squamous cells (case 2), a bacterial protection mechanism that had only been found in urothelial cells [1, 2], indicating that squamous epithelium could be infected; and C) The filamentous morphotype of *E. coli*, which has been associated with phagocytosis evasion by polymorphonuclear cells and an increase in adherence points, can be found in urothelial cells. It is known that, during the pathogenic cycle, filamentous bacteria fragmentate to form short rods that mediate the invasion of superficial cells and contribute to the recurrence of the infection through the invasion of basal urothelial cells and the formation of quiescent intracellular reservoirs associated with chronic infections [2, 8, 9].

A urinary sediment analysis is conventionally used to search for crystals, cylinders, and cells in urine samples. However, it may also be a useful tool in the search of the mechanism of pathogenesis of different microorganism because results, such as intracellular invasion could explain the recurrence of an infection and the failure of an antibiotic treatment. It would be important to determine the prognostic value of IBCs and bacterial morphotypes in urinary tract infections and their clinical significance as well as to explore those intracellular pathways that allow the adaptation of IBCs to squamous cells.

## Data Availability

All the used protocols and materials will be provided after an e-mail to the correspondence author.

## Abbreviations

*E. coli*: *Escherichia coli*
IBC: intracellular bacterial communities
UTI: Urinary tract infections
